# The secretory deficit in islets from *db*/*db* mice is mainly due to a loss of responding beta cells

**DOI:** 10.1007/s00125-014-3226-8

**Published:** 2014-04-06

**Authors:** Oanh H. Do, Jiun T. Low, Herbert Y. Gaisano, Peter Thorn

**Affiliations:** 1 School of Biomedical Sciences, University of Queensland, St Lucia, QLD 4072 Australia; 2 Department of Medicine, University of Toronto, Toronto, ON Canada

**Keywords:** *db*/*db*, Insulin, Islet secretion

## Abstract

**Aims/hypothesis:**

We used the *db*/*db* mouse to determine the nature of the secretory defect in intact islets.

**Methods:**

Glucose tolerance was compared in *db*/*db* and wild-type (WT) mice. Isolated islets were used: to measure insulin secretion and calcium in a two-photon assay of single-insulin-granule fusion; and for immunofluorescence of soluble *N*-ethylmaleimide-sensitive factor attachment proteins (SNAREs).

**Results:**

The 13–18-week-old *db*/*db* mice showed a diabetic phenotype. Isolated *db*/*db* islets showed a 77% reduction in insulin secretion induced by 15 mmol/l glucose and reductions in the amplitude and rise-time of the calcium response to glucose. Ionomycin-induced insulin secretion in WT but not *db*/*db* islets. Immunofluorescence showed an increase in the levels of the SNAREs synaptosomal-associated protein 25 (SNAP25) and vesicle-associated membrane protein 2 (VAMP2) in *db*/*db* islets, but reduced syntaxin-1A. Therefore, *db*/*db* islets have both a compromised calcium response to glucose and a compromised secretory response to calcium. Two-photon microscopy of isolated islets determined the number and distribution of insulin granule exocytic events. Compared with WT, *db*/*db* islets showed far fewer exocytic events (an 83% decline at 15 mmol/l glucose). This decline was due to a 73% loss of responding cells and, in the remaining responsive cells, a 50% loss of exocytic responses per cell. An assay measuring granule re-acidification showed evidence for more recaptured granules in *db*/*db* islets compared with WT.

**Conclusions/interpretation:**

We showed that *db*/*db* islets had a reduced calcium response to glucose and a reduction in syntaxin-1A. Within the *db*/*db* islets, changes were manifest as both a reduction in responding cells and a reduction in fusing insulin granules per cell.

**Electronic supplementary material:**

The online version of this article (doi:10.1007/s00125-014-3226-8) contains peer-reviewed but unedited supplementary material, which is available to authorised users.

## Introduction

The *db*/*db* leptin receptor mutant mouse is a model for type 2 diabetes [1]. The animals gain weight and develop insulin intolerance and reduced insulin secretion [2]. The explanation for this reduction in secretion, as in type 2 diabetes, is still not clear, but may include: loss of islets; loss of beta cells [3]; loss of insulin content [2, 4, 5]; or reduced exocytic capacity. Direct comparison shows the proportionate loss of secretion parallels the loss of insulin content: ∼4.5- to sixfold for both [4, 5]. However, even though content is reduced, there is still more insulin present than that required to maintain normoglycaemia [5]. This suggests defects in insulin secretion are important in the disease; an idea supported by work in humans [6, 7] and in other models of type 2 diabetes, such as the Goto-Kakizaki (GK) rat [8, 9].

The reduced insulin secretion could arise because of defects anywhere along the complex stimulus-secretion cascade. In *db*/*db* mice there is evidence for reduced GLUT2 expression [10, 11], and reduced ATP production [12]. The next step in the cascade is the calcium response. In *db*/*db* mice, both the size [12] and the temporal profile of the calcium response are altered [13]. Most recently, there is evidence, in other disease models, for a mis-positioning of calcium channels relative to the sites of insulin granule fusion [8, 14].

The final step of granule fusion is dependent on the soluble *N*-ethylmaleimide-sensitive factor attachment proteins (SNAREs). In *db*/*db* islets mRNA for synaptosomal-associated protein 25 (SNAP25) and vesicle-associated membrane protein 2 (VAMP2) increase and syntaxin-1A mRNA decreases [15]. This contrasts with the GK rat, in which all these SNAREs decrease [9, 16]. Functional studies in the GK rat show a reduction in insulin granule fusion [17]. It has been suggested that different modes of fusion might be prevalent in models of ‘acute’ disease that use high-glucose or palmitate exposure [18, 19]. Here, transient granule fusion, often termed ‘kiss-and-run’, could preferentially release low molecular mass compounds and only partially release insulin [20]; if this was the prevalent form of granule fusion in disease this could explain the reduction in insulin secretion. However, the relevance of full fusion or kiss-and-run fusion in islets is questioned [21, 22] and possible roles are not known in animal models of disease.

Within the intact islet it is known that the structure and physical relationships between the cells are important for secretion [23], and are factors that change in disease [3, 5]. To understand beta cell secretory function within an islet we have developed a live-cell two-photon assay to measure single-insulin-granule fusion events [24]. We have validated this method on wild-type (WT) islets to prove that it measures insulin granule fusion and that the time course and number of fusion events fully account for the measured amount of insulin secretion [24]. While the method lacks the temporal resolution of capacitance measurement [25], its key advantage is that it records responses from all cells within the two-photon image slice.

Using this method, we show here that the secretory deficit in *db*/*db* islets is primarily due to a loss of responding cells and a reduction in granule (full) fusion in the remaining responsive cells.

## Methods

### Experimental solution

Experiments were performed in an extracellular solution (140 mmol/l NaCl, 5 mmol/l KCl, 1 mmol/l MgCl_2_, 2.5 mmol/l CaCl_2_, 5 mmol/l NaHCO_3_, 5 mmol/l HEPES and glucose [according to test conditions]) adjusted to pH 7.4 with NaOH.

### Cell preparation

Mice (BKS.Cg-Dock7^*m*^ +/+ Lepr^*db*^/J, The Jackson Laboratory, Bar Harbor, Maine, USA) were humanely killed according to local University of Queensland animal ethics procedures (approved by the University of Queensland Anatomical Biosciences Ethics Committee). Isolated mouse pancreatic tissue was prepared by a collagenase (type IV) (Gibco, VIC, Australia) digestion method in Hanks buffer (Sigma-Aldrich, Castle Hill, NSW, Australia), adjusted to pH 7.4 with NaOH. Isolated islets were maintained (37°C, 95/5% air/CO_2_) in RPMI-1640 culture medium (Gibco, Life Technologies, Mulgrave, VIC, Australia) supplemented with 10% FBS (Gibco) and 100 U/ml penicillin/0.1 mg/ml streptomycin (Invitrogen, VIC, Australia).

### Islet imaging

Islets that had been cultured for 2–3 days were pre-incubated in extracellular solution containing 3 mmol/l glucose for 30 min (37°C, 95/5% air/CO_2_) prior to two-photon imaging (see ESM Methods).

### Two-photon imaging

We used a custom-made video-rate two-photon microscope with a ×60 oil immersion objective (NA 1.42, Olympus, Macquarie Park, NSW, Australia), providing an axial resolution (full width, half maximum) of ∼1 μm. We imaged exocytic events using sulforhodamine B (SRB, 800 μmol/l) as a membrane-impermeant fluorescent extracellular marker excited by femtosecond laser pulses at 950 nm, with fluorescence emission detected at 550–650 nm. 8-Hydroxypyrene-1,3,6-trisulfonic acid (HPTS) was excited at 950 nm and emission detected at 420–520 nm.

Images (resolution 10 pixels/μm) were analysed with the Metamorph program (Molecular Devices Corporation, Sunnyvale, CA, USA). Exocytic event kinetics were measured from regions of interest (0.78 μm^2^, 78 pixels) centred over individual granules. Traces were rejected if extensive movement was observed.

### Glucose tolerance test

After an overnight fast (17:00 to 9:00 hours), fasting blood glucose concentrations were obtained by the Accu-check Active (Roche, Castle Hill, NSW, Australia) glucometer with a small drop of blood (∼5 μl) from the tail tip. Then glucose 25% (wt/vol.) was injected intraperitoneally (1 g/kg) and blood samples were taken at 15, 30, 60, 90 and 120 min and glucose concentrations measured.

### Immunofluorescence

Isolated islets were fixed in 4% paraformaldehyde, permeabilised with Triton X-100 or saponin and blocked with donkey serum prior to incubation with primary antibodies (see ESM Methods).

### Insulin assay

We used homogeneous time-resolved fluorescence (HTRF) with reagents supplied by Cisbio (HTRF Insulin Kit [number 62INSPEB], Arundel, QLD, Australia) to measure islet insulin secretion (see ESM Methods).

### Calcium measurement

For intracellular calcium measurement, we used the ratiometric calcium indicator Fura 2-AM (Molecular Probes, Life Technologies, see ESM Methods).

### Statistical analyses

Data are presented as mean ± SEM. Statistical analyses were performed using Microsoft Excel and GraphPad Prism. Data sets were subjected to a Student’s *t* test; ^*^ *p* < 0.05, ^**^ *p* < 0.01 and ^***^ *p* < 0.001. Islets from at least four animals were used in each experiment.

## Results

### The db/db mice have a diabetic phenotype

The B6.BKS(D)-Lepr^*db*^/^*db*^ mice became significantly heavier than their WT littermates (ESM Fig. 1a, *n* = 38 mice, 13–18 weeks of age, one-way ANOVA *p* < 0.0001). At 13–18 weeks the *db*/*db* mice showed significant differences in glucose tolerance tests (GTTs), indicative of the development of diabetes (ESM Fig. 1b, c; *n* = 38 mice, one-way ANOVA *p* < 0.0001). These changes in the *db*/*db* animals are due to both peripheral insulin resistance [2] and glucose insensitivity of insulin secretion from the islets [2, 5].

### Insulin secretion is reduced in db/db islets

**Fig. 1:**
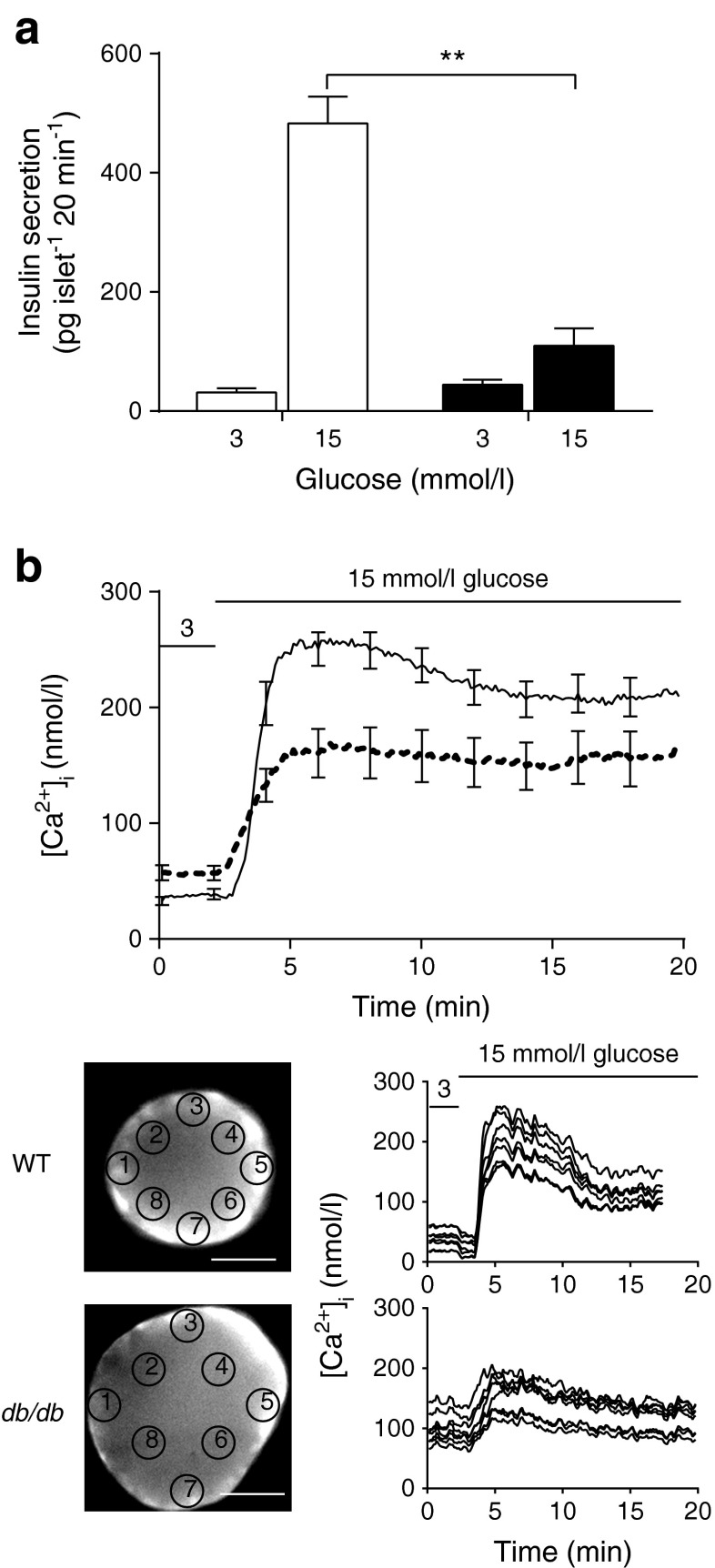
Insulin secretion in *db*/*db* islets is decreased. (**a**) A static insulin secretion assay, performed over 20 min, shows that the glucose-dependent increase in insulin secretion in WT islets (white bars) is almost completely lost in *db*/*db* islets (black bars), *n* = 10–12 mice. (**b**) The calcium response to glucose is smaller and slower in the *db*/*db* islets (dotted line) compared with WT islets (solid line) (NB individual data were aligned to the rising phase). For both WT and *db*/*db* islets, the calcium response is relatively uniform across individual islets, *n* = 20–30 islets. Scale bar 50 μm. ^**^ *p* < 0.01

Islets were isolated from WT and *db*/*db* mice, cultured for 2–3 days and tested for glucose-dependent insulin secretion. We applied 15 mmol/l glucose for 20 min and measured the secreted insulin. Islets from *db*/*db* mice had a significantly reduced response (Fig. 1a; *n* = 22 mice, Student’s *t* test, *p* < 0.01). Together, the weight gain, decreased GTT and reduction in insulin secretion are consistent with previous results [2, 4, 26].

We next focused on studying the two distal steps of the stimulus-secretion cascade: the rise in cytosolic calcium and the exocytic fusion of insulin granules.

### Calcium responses are attenuated in db/db islets

Fura 2-AM-loaded WT islets were stimulated with 15 mmol/l glucose. The calibrated fluorescent signals showed a rapid increase in cytosolic calcium from 50 to ∼250 nmol/l (Fig. 1b) that appeared relatively uniform across individual islets. In contrast, *db*/*db* islets had significantly higher resting calcium levels (WT 37.6 nmol/l vs *db*/*db* 56.8 nmol/l, *p* < 0.05) and a significantly lower rate of increase in calcium (WT 3.48 nmol l^−1^ s^−1^ vs *db*/*db* 0.95 nmol l^−1^ s^−1^, *p* < 0.001) and peak response to glucose (Fig. 1b, WT 281.7 nmol/l vs *db*/*db* 193.0 nmol/l, *p* < 0.01); again, the calcium response was relatively uniform across the islets.

### The final stages of granule fusion are impaired in db/db islets

**Fig. 2:**
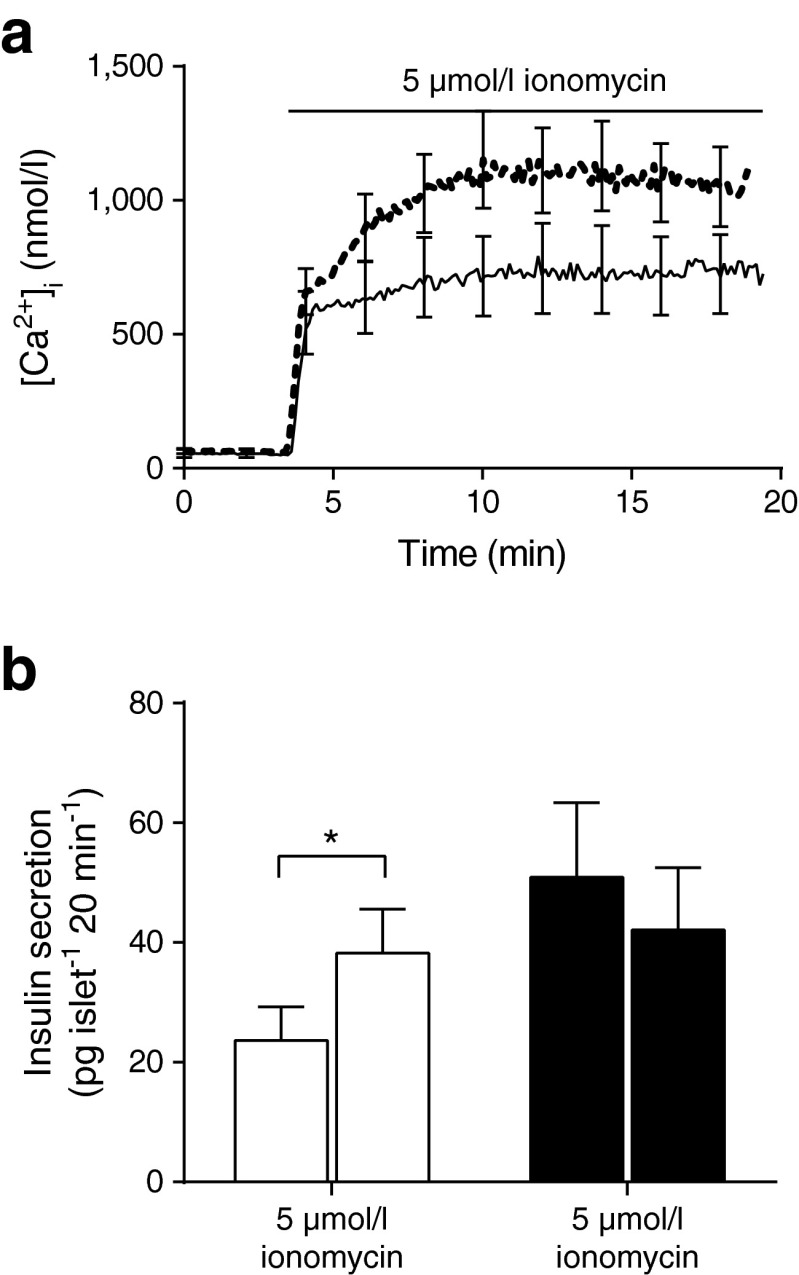
Ionomycin fails to induce insulin secretion in *db*/*db* islets. (**a**) The calcium response to ionomycin, measured as calibrated Fura 2-AM fluorescence, in WT (solid line) and *db*/*db* islets (dotted line) have similar kinetics, but *db*/*db* islets have a bigger magnitude. (**b**) A static insulin assay, performed over 20 min, shows WT islets (white bars) respond to ionomycin with a 1.6-fold increase in insulin secretion whereas the *db*/*db* islets (black bars) show no response, *n* = 11–12 mice. ^*^ *p* < 0.05

To test for a deficit in the last stage of the stimulus-secretion cascade, we artificially raised cytosolic calcium using 5 μmol/l ionomycin. Ionomycin induced large cytosolic calcium responses in both WT and *db*/*db* islets (Fig. 2a, *n* = 14 mice). However, ionomycin-induced insulin secretion was observed only in WT islets (Fig. 2b, *n* = 23 mice). This shows that the secretory response to a calcium signal is reduced in *db*/*db* islets and demonstrates a specific defect in granule fusion.

**Fig. 3:**
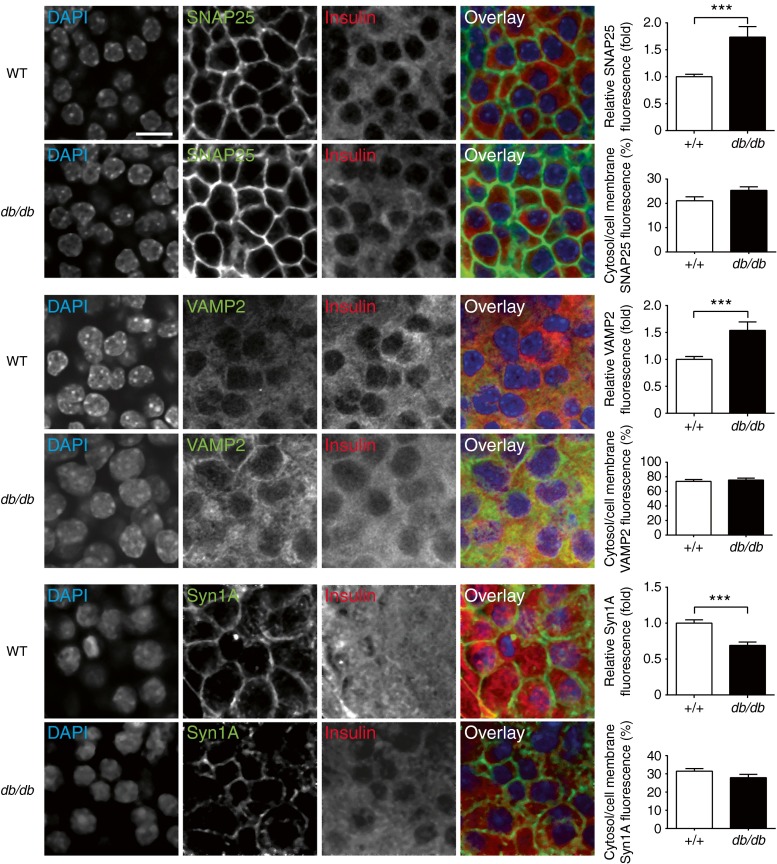
SNARE protein distribution and expression are altered in *db*/*db* islets. Immunofluorescence of fixed islets shows insulin staining (red) SNAP25, VAMP2 and syntaxin-1A in beta cells. The histograms show average fluorescence intensity changes in *db*/*db* islets normalised to WT and % of fluorescence intensity in cytosol regions compared with plasma membrane regions in *db*/*db* and WT islets, *n* > 4 mice, 15–19 islets. Scale bar 10 μm. ^***^ *p* < 0.001. Syn1A, syntaxin-1A

Immunofluorescence was then used to determine the relative expression and distribution of SNAREs in the islets. Based on insulin immunostaining, almost all the cells in the core of the islets were beta cells (Fig. 3). SNAP25 and syntaxin-1A localised to the beta cell membrane. And VAMP2, the granule SNARE, was found within the cell. Consistent with previous measurement of mRNA levels [15] we show that levels of SNAP25 and VAMP2 proteins were increased in the *db*/*db* islets but syntaxin-1A was reduced (Fig. 3).

Taken together, our data indicate that the *db*/*db* islets have a reduced calcium response to glucose and a compromised secretory response that is possibly due to a lower level of syntaxin-1A.

### Specific measurement of single-granule-fusion events in islets

To understand how these secretory defects are manifest in an islet we exploited a two-photon assay to measure single-granule-fusion events within the core of intact islets of Langerhans [24], where almost all the cells in rodent islets are beta cells. This assay tracks the entry of extracellular dye as each insulin granule fuses; it has been extensively validated in a previous paper [24].

**Fig. 4:**
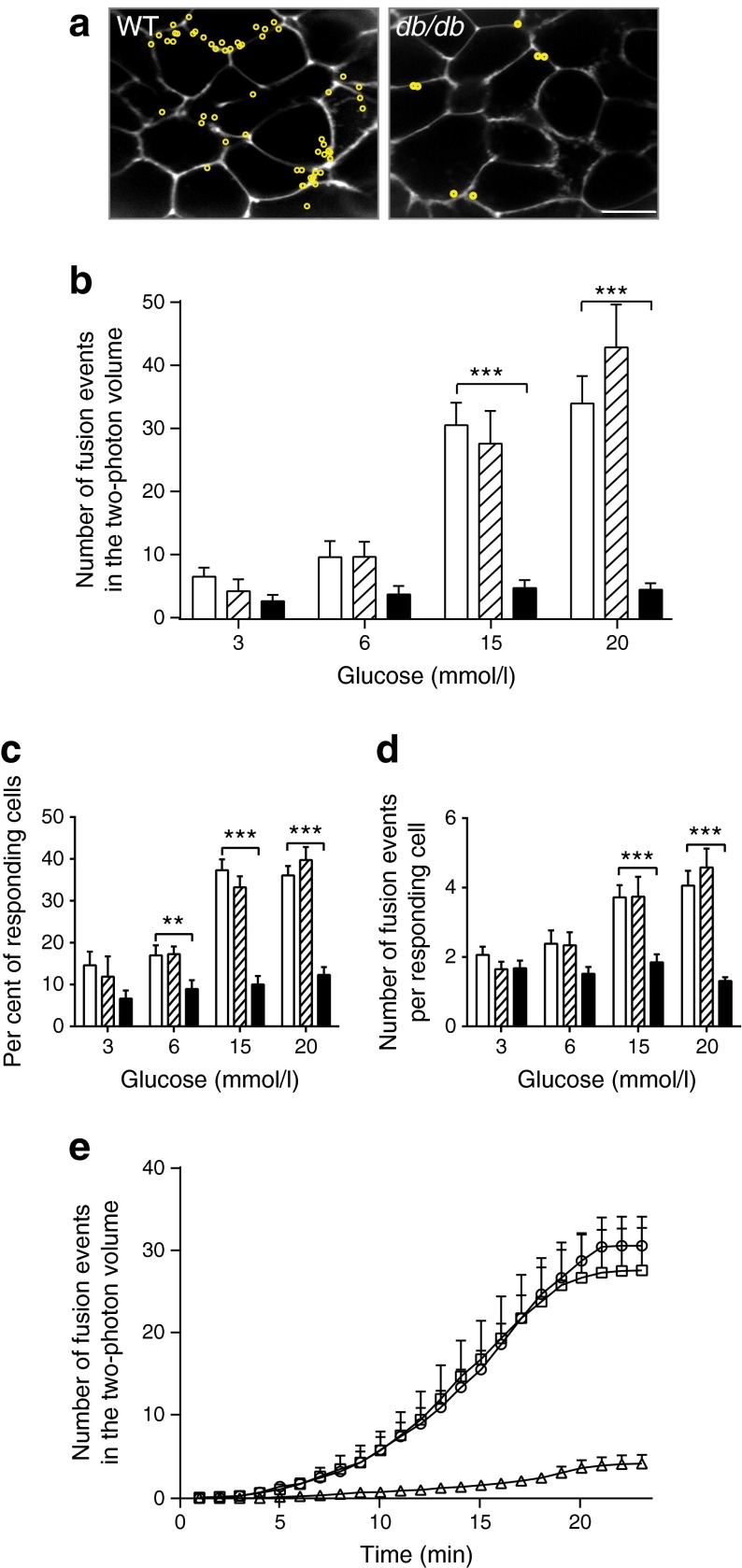
Within intact islets from *db*/*db* mice, beta cells have fewer insulin granule fusion events. (**a**) Typical two-photon cross sections taken across intact islets include many cells. Each granule fusion event over a 20 min time period is identified (as shown in ESM Fig. 2) and its position marked as a yellow circle. Scale bar 10 μm. (**b**) Quantification of the numbers of granule fusion events (in the two-photon volume, over 20 min), across a range of glucose concentrations, shows a significant reduction in granule fusion in *db*/*db* beta cells. The reduction in fusion events observed occurs because of both (**c**) a decrease in the number of responding cells and (**d**) a decrease in the number of fusion events per responding cell. (**e**) Cumulative plots of insulin granule fusion events over time, in response to 15 mmol/l glucose. *n* = 4–9 mice and 175 islets; *p* values from ANOVA analysis: ^**^ *p* < 0.01 and ^***^ *p* < 0.001. In b–d: white bars, WT; hatched bars, *db*/+; black bars, *db*/*db*. In e: circles, WT; squares, *db*/+; and triangles, *db*/*db*

Isolated islets were bathed in extracellular solution containing the fluorescent dye SRB and imaged with two-photon microscopy [24, 27]. The concentration of extracellular glucose was changed and the total numbers of fusion events occurring within the two-photon sample volume (∼40 × 50 × 1 μm) were recorded over 20 min. ESM Fig. 2 shows a single beta cell within an islet, where 15 mmol/l glucose-induced five-granule-fusion events, with each event showing a characteristic rapid rise in fluorescence after fusion and then a slower decay back to baseline fluorescence. Control WT islets showed a glucose-dose-dependent increase in the number of fusion events, consistent with our previous data (Fig. 4a, b; *n* = 177 islets, 21 mice [24]). In contrast, islets from *db*/*db* animals showed significantly lower numbers of fusion events induced by glucose (Fig. 4, one-way ANOVA: 3 mmol/l, *p* = 0.11; 6 mmol/l, *p* = 0.061; 15 mmol/l, *p* = 1.23 × 10^−9^; 20 mmol/l *p* = 8.24 × 10^−8^, e.g. an 83% loss at 15 mmol/l). Further analysis demonstrates that, in response to 15 mmol/l glucose, the number of responding cells in *db*/*db* islets was reduced by 73% and the number of granule fusion events in the remaining responsive cells was reduced by 50% (Fig. 4c, ANOVA for number of responding cells: 3 mmol/l, *p* = 0.15; 6 mmol/l, *p* = 0.0096; 15 mmol/l, *p* = 5.83 × 10^−12^; 20 mmol/l, *p* = 1.8 × 10^−10^). ANOVA for the number of fusion events per responding cell: 3 mmol/l, *p* = 0.38; 6 mmol/l, *p* = 0.11; 15 mmol/l, *p* = 0.0001; 20 mmol/l, *p* = 9.12 × 10^−8^. While we cannot identify cell type in this assay, the measured perimeters of the responsive cells in control and *db*/*db* islets were the same (ESM Fig. 3), suggesting both are beta cells as the other possible cell types are a different size [25].

The time course of the cumulative exocytic responses in WT islets shows an increase, up to a plateau, in response to 15 mmol/l glucose (Fig. 4d). In contrast, *db*/*db* islets showed a steady and small exocytic response.

These data show *db*/*db* beta cells have dramatically reduced numbers of exocytic fusion events. However, there may be additional variables of fusion that differ in *db*/*db* cells. For example, the duration of granule fusion and the behaviour of the granule after fusion could be different and both could have an influence on the amount of insulin secreted [20, 28].

### Exocytic lifetimes and granule fusion characteristics are not different in db/db beta cells

**Fig. 5:**
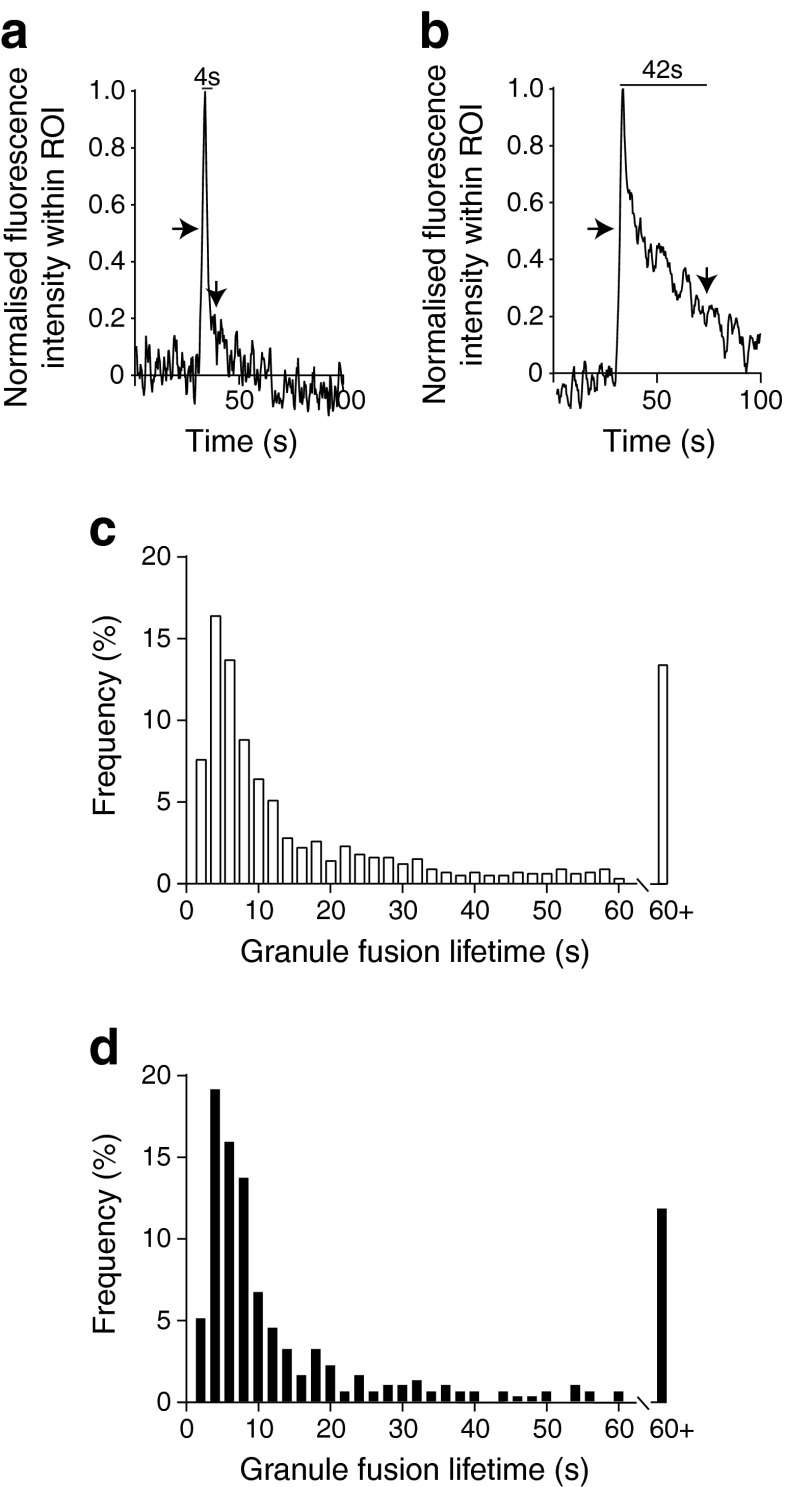
Insulin granule fusion lifetimes are not changed in *db*/*db* cells. Examples of (**a**) short and (**b**) longer granule lifetimes as identified by the average fluorescence signal from regions of interest (ROIs) placed over each individual fusion event. Arrows show the start and end of the event. (**c**, **d**) Frequency graphs of the binned granule lifetimes; WT (**c**) and *db*/*db* islets (**d**) have similar distributions; *n* = 4–9 mice, total of 1,057 events

We quantified granule fluorescence lifetimes, arbitrarily defining the start of the event as where the fluorescence rose to the half maximal fluorescence intensity and the end of the event when the signal dropped to below the two standard deviations above the baseline fluorescence noise (Fig. 5a, start and end indicated by arrows). Frequency–lifetime histograms show a model value of ∼4 s (Fig. 5c and d; WT, *n* = 743 events; *db*/*db*, *n* = 314 events), with no evidence for distinct populations of lifetimes, and no differences between *db*/*db* and WT (Kolmogorov–Smirnov test between WT and *db*/*db*, *p* = 0.422).

**Fig. 6:**
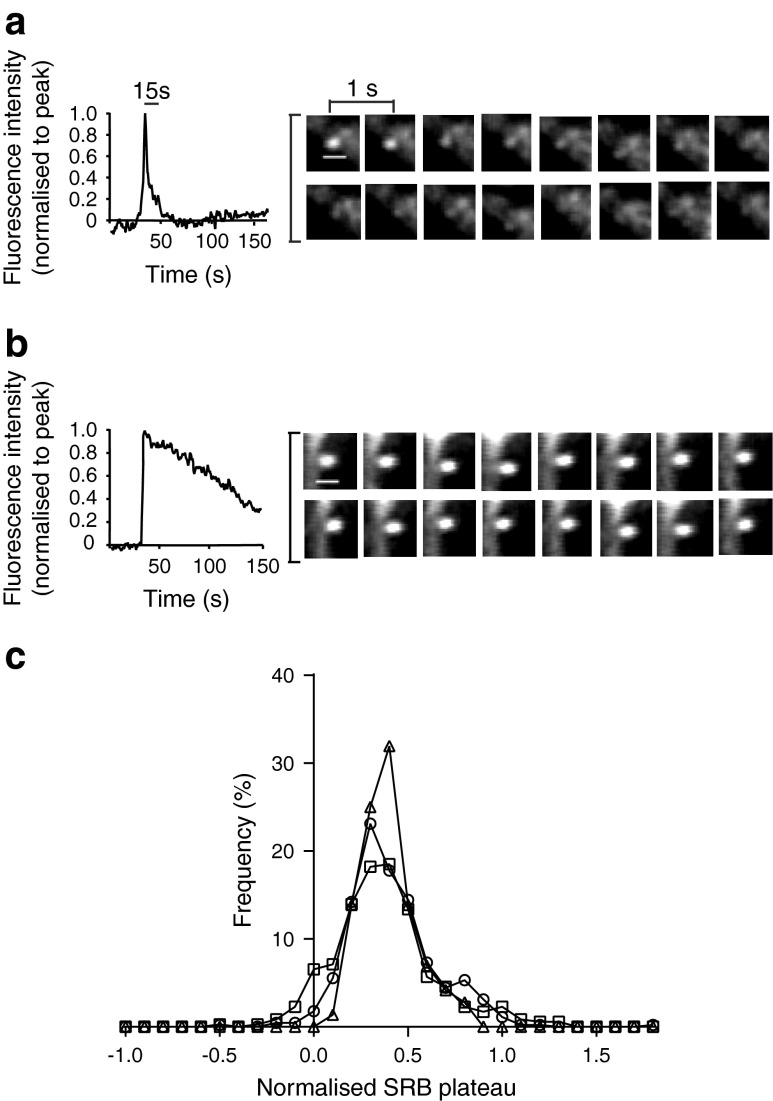
The granule fluorescence plateaus are not changed in *db*/*db* cells. Two examples of single-granule-fusion events: (**a**) a low fluorescence plateau (fluorescence returns to background levels) (plateau = 0.09); and (**b**) a high fluorescence plateau (plateau = 0.87). In each case the mean fluorescence changes within a region of interest placed over the granule fusion site is shown, as are images taken at 1 s intervals over the duration of the fusion event. Scale bar 1 μm. (**c**) Graphs of frequency plotted against fluorescence (SRB) plateau (normalised to the peak fluorescence) are similar for WT (circles), *db*/+ (squares) and *db*/*db* islets (triangles)

Another variable is the fluorescence intensity post-fusion, which is different for each granule (Fig. 6). In some fusion events the fluorescence stays high; we have previously shown that this reflects the retention of granule content (and dye) within the granule lumen [29]. Figure 6a and b show two examples of the fluorescence profile over time from two different exocytic events. In one event there is a rapid loss of fluorescence, while in the other the fluorescence signal is maintained at a high intensity. To quantify this for each granule fusion event, we measured the fluorescence intensity 15 s after the peak fluorescence, normalised this fluorescence to the peak fluorescence and plotted out a frequency graph (Fig. 6c). The modal value of this fluorescence signal was around 0.3 (i.e. a plateau level of 30% of the peak fluorescence) and the distribution of values did not differ between WT, *db*/+ and *db*/*db* islets (Kolmogorov–Smirnov test between WT and *db*/*db*, *p* = 0.514).

We conclude that, using our assays, the insulin granule fusion events in *db*/*db* beta cells are indistinguishable from those in WT cells.

### Granule endocytosis

After fusion, granule fluorescence persisted for a short period of time (see Fig. 5) and was then lost, which we interpret as a collapse of the granule into the cell membrane. However, another alternative granule behaviour is that the fusion pore can close while the granule is at the cell membrane, sometime called ‘kiss-and-run’, which is well documented in beta cells [20, 30]. After fusion pore closure the granule lumen rapidly acidifies because of the action of the granular vacuolar (v)ATPase [31].

**Fig. 7:**
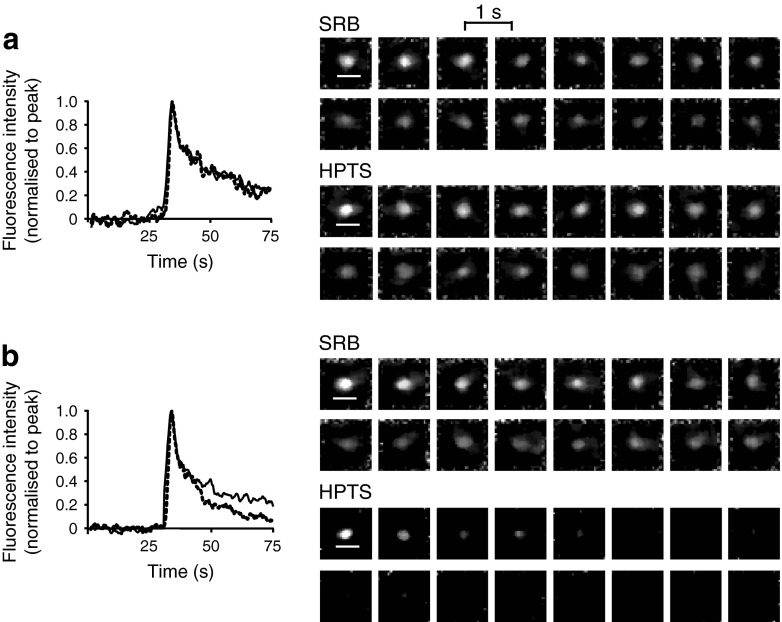
‘Kiss-and-run’ granule recapture is increased in the *db*/*db* cells. Two extracellular dyes, SRB (pH insensitive) (solid lines) and HPTS (dashed lines) both enter each fusing granule. (**a**) For most granule fusion events the average fluorescence intensity changes are similar for both dyes. (**b**) In contrast, some granule fusion events show a selective decrease in the HPTS signal where it is quenched by acidification. Scale bars 1 μm; *n* = 4 WT and *n* = 4 *db*/*db* mice; total of 580 events

To detect this pH shift we added a pH-sensitive dye (HPTS) to the extracellular solution (in addition to SRB, which is pH insensitive) and tracked the fluorescence changes after granule fusion [32]. Acidification reduces the HPTS fluorescence [33], and a different fluorescence signal between the two dyes indicates granule recapture (Fig. 7). To quantify these changes we define a ‘recaptured’ granule as one where, at 15 s after the peak, the HPTS had dropped by more than 20% of the SRB signal. This quantification demonstrates that the proportion of granules that acidified was small but interestingly there were significantly more recaptured granules (×1.75 increase) in the *db*/*db* islets (Table 1; Student’s *t* test, *p* = 0.022). We conclude that transient fusion (kiss-and-run) is more common in the *db*/*db* islets.

**Table 1:** Proportions of acidifying (recaptured) granules in WT and *db*/*db* islets

Islet	Total exocytic events	Number (%) of re-acidified events
WT	408	27 (6.6)
*db*/*db*	172	20 (11.6)

## Discussion

The basis of the insulin secretory effect in type 2 diabetes is still not fully understood. In principle, changes could occur in the number of responding islets, number of beta cells or in the secretory function of individual beta cells. Our data provide clear evidence for a reduction in the number of responding cells and a decrease in granule fusion.

### Decrease in the number of responding cells

We showed that the number of glucose-responsive cells in the *db*/*db* islets was reduced by 73%, indicating this is the major factor that explains the loss of insulin secretion from islets. This pattern of loss of cell responsiveness is the likely outcome of intrinsic defects in both intracellular beta cell morphology [5] and function, in part caused by endoplasmic reticulum stress [34], that result in apoptosis and dedifferentiation [35]. There is also likely to be a contribution from perturbation of intercellular communication between the islet cells [36], much of which is unknown and requires further study with approaches like ours to measure cell activity within intact islets. The data we show are the first to characterise the functional outcomes of these cellular changes in diseased islets and demonstrate that complete loss of beta cell function is the most significant factor contributing to the overall loss of insulin secretion from islets.

### Decrease in single-cell secretion

We further show that in the cells that remain responsive there is a 50% loss of exocytic fusion. Our two-photon assay unequivocally identifies loss of the number of granule fusion events as the manifestation of this secretory defect. There are two other studies that have looked at insulin granule fusion in models of disease. Using the GK rat model, a study focusing on granule motion before fusion showed a reduction in fusion events [17]. The second study used cultured single cells to show incubation in high glucose selectively reduced full-fusion events compared with transient fusion [19]. These studies are limited to single-cell work, but both agree with our finding that loss of secretion in the damaged beta cell is characterised by a loss of granule fusion. Where our findings differ is that we see no evidence that transient fusion is a significant contributor to the deficit in insulin secretion and our method records the relative contribution of the loss of responsive cells.

### Secretory defect

In the beta cells that poorly secrete, our data suggest some important factors that might explain this reduction in secretory competence.

First, we show changes in calcium signalling that lead to an increased resting calcium and a decreased response to glucose. These observations are consistent with a single-cell study on *db*/*db* beta cells [37], but in intact *db*/*db* islets there is evidence for larger [13] and smaller [12] calcium responses. These differences may reflect stages in disease progression in individual animals, with the larger responses (also seen in the GK rat model [8]) representing an early stage of compensatory upregulation of responsiveness. To date, we have been unable to reliably measure calcium and exocytosis simultaneously in single cells within the islet and therefore cannot directly relate the changed calcium responses to secretory output. However, it seems reasonable that the reduced rate of increase in the calcium signal and the reduced amplitude would lead to a decreased exocytic response. Interestingly, the increased basal calcium levels we observed could explain the increased basal insulin secretion that we (Fig. 2) and others [13] see in the *db*/*db* islets. Our whole-islet calcium measurement method does not spatially or temporally resolve single-cell responses. However, our observed slowed rate of rise of the *db/db* calcium response (Fig. 1b) is consistent with studies that indicate displacement of calcium channels around the cells and away from the sites of exocytosis [14]. What our work with ionomycin clearly indicates is that, on top of a reduced glucose-induced calcium response, there is also a specific loss of exocytic competence in the *db*/*db* beta cells.

The second factor we identify are changes in the expression of SNARE proteins which could contribute to this loss of secretory competence. Interestingly, the increase in SNAP25 and VAMP2 and decrease in syntaxin-1A expression is the same pattern as previously observed in *db*/*db* mice using quantitative PCR [15], which demonstrates a specificity to the cell damage. The changes in SNARE expression are consistent with previous reports [9, 16, 38] and with evidence that manipulation of SNARE expression changes insulin secretion [39]. However, if SNARE reduction was the only defect, it should lead to granule accumulation, something observed in other cell types with a SNARE defect [32]. In fact, in *db*/*db* islets the granule density (per unit cell volume) is approximately half that of WT [40], indicating that there must be additional factors that are changed in disease. This reduction in granule numbers alone does not explain the ∼80% reduction in granule fusion we observe (Fig. 4) and leads us to conclude that the combination of loss of granules, changes in SNARE expression and changes in the calcium signal all contribute to the secretory defect. Such a multifaceted phenotype is consistent with gross cellular changes due, for example, to dedifferentiation [35].

### Changes in insulin granule fusion behaviour

Our work shows that granule lifetimes and the granule fluorescence plateaus are not changed, indicating that the essential exocytic mechanisms are not altered in disease. The increase in kiss-and-run events we observed could be due to the changed expression of SNAREs. The increase in recaptured granules cannot explain the loss of insulin secretion but, instead, could be part of the mechanism of disease. For example, it has been observed that diseased beta cells contain many granules (or at least granule-like objects) in which the insulin crystal is absent [40]. It has been speculated this is due to misprocessing of granule cargo [40], an idea supported by work on mutations in the zinc transporter [41]. But the increase in kiss-and-run events that we observed would also be consistent with the appearance of ‘empty’ vesicles. If these ‘empty’ vesicles could not fuse again then they could form a barrier to exocytosis of normal vesicles and thus contribute to disease progression.

### Conclusion

Among the possible explanations of the diabetic phenotype of *db*/*db* mice we here show that the major factors are the loss of responding cells and the loss of the number of fusing insulin granules.

## Electronic supplementary material

Below is the link to the electronic supplementary material.

ESM Methods (PDF 115 kb)

ESM Fig. 1 (PDF 17 kb)

ESM Fig. 2 (PDF 28 kb)

ESM Fig. 3 (PDF 14 kb)

## Abbreviations

GK: Goto-Kakizaki
GTT: Glucose tolerance test
HPTS: 8-Hydroxypyrene-1,3,6-trisulfonic acid
SNAP25: Synaptosomal-associated protein 25
SNARE: Soluble *N*-ethylmaleimide-sensitive factor attachment proteins
SRB: Sulforhodamine B
VAMP2: Vesicle-associated membrane protein 2
WT: Wild-type
