# Supplementary material for: The secretory deficit in islets from db/db mice is mainly due to a loss of responding beta cells
Source: Diabetologia. 2014 Apr 6;57(7):1400–9. doi: 10.1007/s00125-014-3226-8 (PMC4052007; doi:10.1007/s00125-014-3226-8)
Supplement: Supplementary file 1 — (PDF 115 kb) [file 125_2014_3226_MOESM1_ESM.pdf]

ELECTRONIC SUPPLEMENTARY MATERIAL

ESM Methods

Islet imaging Two-photon imaging was performed at 34°C, with exocytic events recorded as the entry of extracellular dye into each fused granule. The image plane was consistently set at three cell thicknesses above the bottom of the cover-slip (ie in to the islet) where the vast majority of cells in a rodent islet are beta cells. This indirect assay has advantages. It is a sensitive assay capable of recording single exocytic events in our system at a rate of 4 frames per second. It spatially and temporally distinguishes exocytic events within single cells within an intact islet. It does not involve labelling of heterologously expressed proteins or loading of tracers into the cell [22,24].

Immunofluorescence For primary antibodies, we used mouse anti-SNAP25 (Synaptic Systems 111 011); mouse anti-VAMP2 (Synaptic Systems 104 211); mouse anti-Syntaxin-1A (Synaptic Systems 1110 111); rabbit anti-insulin (Genesearch 3014S). Secondary antibodies were highly cross absorbed donkey or goat antibodies (Invitrogen) labelled with Alexa 488 or Alexa 546. All were used at a 1/200 dilution. DAPI (Sigma, 100 ng/ml final concentration).

Insulin assay To acquire insulin samples, pancreatic mouse islets were preincubated in a Na-rich extracellular solution containing 3 mmol/l glucose for 30 minutes. Groups of four islets were then transferred to individual eppendorf tubes containing Na-rich extracellular solution, 0.2% bovine serum albumin (Sigma Aldrich), glucose (3 mmol/l, 15 mmol/l) or ionomycin (5 µm) (Sigma Aldrich) and incubated (37°C, 95/5% air/CO_2) for 20 minutes. Supernatants from each tube were collected and used for HTRF as per Cisbio insulin kit instructions. Briefly, for each sample, 10 µl of islet supernatant, 5 µl of anti-insulin Europium cryptate and 5 µl of anti-insulin XL665 were sequentially added to a 384 well plate (Perkin Elmer Proxiplate 384-Plus, cat. no. 6008280). The plate was incubated on lab bench for 24 hours then read on an Infinite 200 PRO (Tecan group Ltd.) machine. Insulin concentrations (ng/ml) for each well were determined using insulin standard curves.

Calcium measurement After 2-3 days in culture, islets were loaded with 4 µm Fura-2-AM (Invitrogen, Victoria, Australia), 0.02% pluronic acid (Sigma Aldrich) and incubated (37^0C, 95/5% air/CO_2) for 30 minutes. After labelling, islets were washed and incubated in a Na-rich extracellular buffer containing 3 mmol/l glucose for an additional 5 minutes. Then single islets were transferred to a small volume chamber mounted on the stage of an inverted fluorescence microscope with a 40x objective. The cells were alternatingly excited at 340 and 380 nm, and the ratio was collected at 490 nm.
