# Supplementary material for: The secretory deficit in islets from db/db mice is mainly due to a loss of responding beta cells
Source: Diabetologia. 2014 Apr 6;57(7):1400–9. doi: 10.1007/s00125-014-3226-8 (PMC4052007; doi:10.1007/s00125-014-3226-8)
Supplement: Supplementary file 2 — (PDF 17 kb) [file 125_2014_3226_MOESM2_ESM.pdf]

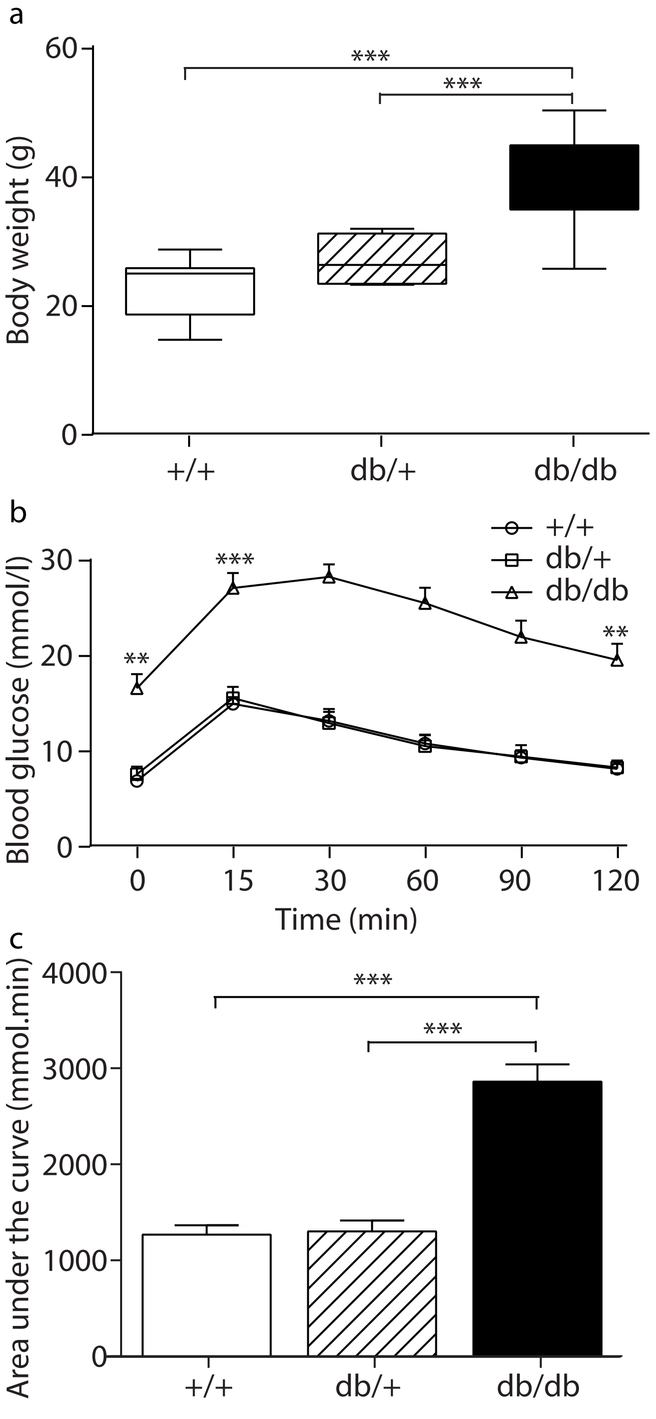

ESM Fig. 1 Db/db mice show a diabetic phenotype. (a) shows the increase in body weight in littermates of the wild-type, db/+ and db/db animals measured at 12 weeks. (b and c) significant changes were observed in the GTTs and integrated AUCs from the different mice (aged between 13 and 18 weeks, n=4-27 mice). Asterisks indicate p values from Student's t test analysis, $^{**}p<0.01$, $^{***}p<0.001$.
