# Supplementary material for: The secretory deficit in islets from db/db mice is mainly due to a loss of responding beta cells
Source: Diabetologia. 2014 Apr 6;57(7):1400–9. doi: 10.1007/s00125-014-3226-8 (PMC4052007; doi:10.1007/s00125-014-3226-8)
Supplement: Supplementary file 3 — (PDF 28 kb) [file 125_2014_3226_MOESM3_ESM.pdf]

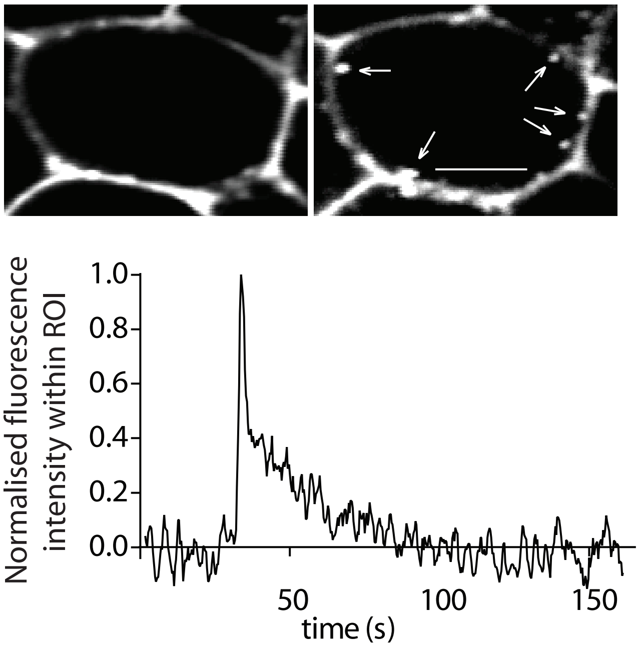

ESM Fig. 2 Extracellular fluorescent dye enters each insulin granule as it fuses with the cell membrane. The images show a wild-type beta cell before stimulation (left) that is within the islet and is outlined by extracellular dye (SRB). During glucose stimulation exocytosis around the periphery of the cell is identified as the entry of dye into each fusing granule (arrows) here shown as a maximum projection image of a series of images captured during the response. A typical graph of average fluorescence over time, from a region of interest (ROI) placed over the area of single fusion event is shown. Scale bar 5 µm.
