# Supplementary material for: The secretory deficit in islets from db/db mice is mainly due to a loss of responding beta cells
Source: Diabetologia. 2014 Apr 6;57(7):1400–9. doi: 10.1007/s00125-014-3226-8 (PMC4052007; doi:10.1007/s00125-014-3226-8)
Supplement: Supplementary file 4 — (PDF 14 kb) [file 125_2014_3226_MOESM4_ESM.pdf]

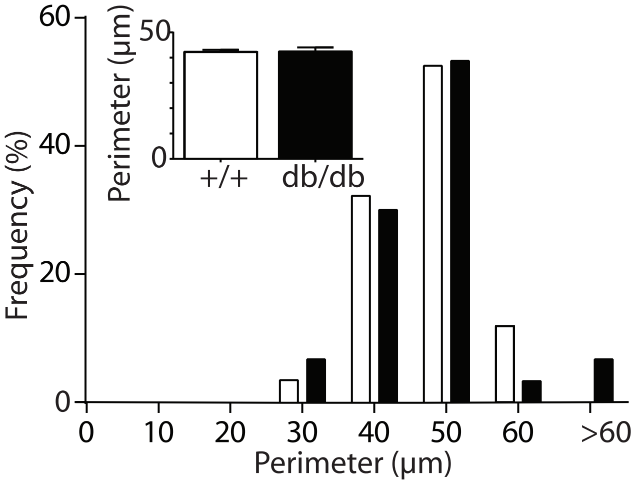

ESM Fig. 3 The size of the responding cells in wild type and db/db islets is the same. We measured cell perimeter in the two-photon images in the cells that responded to a glucose challenge. Neither the length of the perimeter, nor the frequency distributions of the perimeters were different.
